# Effects of replacing zinc oxide with different levels of zinc lactate on growth performance, serum indexes, intestinal health and gut microbiota in weaned piglets

**DOI:** 10.3389/fmicb.2025.1622700

**Published:** 2025-07-11

**Authors:** Fawen Dai, Fei Zhao, Xia Huang, Muqu Jin, Qin Zhou, Tao Lin, Jianjun Zuo, Yongwen Zhu

**Affiliations:** ^1^College of Life Science, Leshan Normal University, Leshan, China; ^2^Provincial Engineering and Technology Research Center for Innovative Development of Bamboo Fiber Nutrition, Leshan, China; ^3^Key Laboratory of Bamboo Pest Control and Resource Development, Leshan, China; ^4^Hunan DeBon Bio-tech Co., Ltd., Hengyang, China; ^5^College of Animal Science, South China Agricultural University, Guangzhou, China

**Keywords:** weaned piglets, zinc lactate, zinc oxide, growth performance, antioxidant capacity, intestinal health, cecal microbiota

## Abstract

**Introduction:**

This study evaluated the efficacy of substituting zinc oxide (ZnO) with varying levels of zinc lactate (ZnL) in weaned piglets.

**Methods:**

A total of 128 Duroc × Landrace × Yorkshire (DLY) weaned piglets (7.31 ± 0.25 kg) were randomly divided into 4 dietary groups for 18 days (*n* = 4 in each group): CON-(basal diet, no ZnO); CON+, basal + 1500 ppm zinc with ZnO; TRE1 (basal + 600 ppm zinc with ZnL); TRE2 (basal diet + 800 ppm zinc with ZnL).

**Results:**

TRE2 achieved comparable growth performance (average daily gain, average daily feed intake, and feed intake to gain ratio) to CON+ (*P* > 0.05), while both showed significantly lower average diarrhea rates than CON- (*P* < 0.05). The skin redness scores in TRE1 (*P* > 0.05) and TRE2 (*P* > 0.10) were higher than CON+ on day 7 of the experiment. Supplementation with 800 ppm ZnL enhanced antioxidant capacity, increasing serum glutathione peroxidase (GPx), superoxide dismutase (SOD), copper-zinc superoxide dismutase (Cu/Zn-SOD) activities compared to CON- (*P* < 0.05), matching CON+ levels. TRE2 significantly increased the duodenal and jejunal villus height compared to CON- (*P* < 0.05), similar to CON+, and elevated duodenal/ileal mRNA expression of Claudin-1 compared to both controls (*P* > 0.05). Cecal microbiota analysis (16S rRNA) revealed that CON+ and TRE2 significantly reduced richness indices (Ace, Chao, and Sobs; *P* < 0.05) and increased the Simpson diversity index (*P* < 0.05) versus CON-. Compared with CON-, CON+ significantly increased proportions of genera *Clostridium_sensu_stricto_1* and *Streptococcus* (*P* < 0.05) while decreasing *[Eubacterium]_ruminantium_group* (*P* < 0.05). TRE2 also had significantly reduced proportions of *Christensenellaceae_R-7_group*, *Ruminococcus and [Eubacterium]_ruminantium_group* (*P* < 0.05). Spearman correlation analysis revealed a significantly positive correlation between *Clostridium_sensu_stricto_1* and serum SOD (*P* < 0.01), as well as between *[Ruminococcus]_gauvreauii_group* and GPx (*P* < 0.05). In contrast, *Clostridium_sensu_stricto_1* was significantly negatively correlated with serum malondialdehyde (*P* < 0.01).

**Discussion:**

In conclusion, dietary supplementation of 800 ppm zinc from ZnL achieved equivalent effects to 1500 ppm ZnO in enhancing antioxidant capacity and gut health by regulating the gut microbiota, providing an effective zinc reduction strategy for weaned piglet nutrition.

## Introduction

Weaning is a critical phase in pig production, often associated with oxidative stress and injury in piglets due to a combination of physiological, environmental, and social stressors (Yin et al., 2014), which contribute to the increased mortality rates during the weaning period (Su et al., 2022). To mitigate these adverse effects, high dietary levels of ZnO have been extensively utilized in weaned piglet diets owing to their antimicrobial, antioxidant, and growth-promoting properties (Biswas et al., 2024; Peng et al., 2019; Xiao et al., 2023). However, the application of high-dose ZnO raises significant concerns, including environmental contamination, zinc accumulation in animal tissues, and the potential emergence of antimicrobial resistance (Dalto et al., 2023a; Ekhlas et al., 2023). In response to these issues, this regulatory shift has intensified the need to identify effective and sustainable alternatives to ZnO in weaned piglet diets in the European Union implemented in pig feed (Pejsak et al., 2023). Therefore, numerous studies have been conducted to explore the potential of low-dose novel zinc sources as alternatives to pharmacological doses of conventional ZnO in weaned piglets. These include sodium alginate-coated nano zinc oxide (Xiao et al., 2023), tetrabasic zinc chloride (Peng et al., 2024), protein-chelated zinc (Zhang et al., 2022), palygorskite clay-adsorbed nano-ZnO (Yu et al., 2022), and zinc aspartic acid chelate (Biswas et al., 2024). The effective addition level of the ZnO substitute in the above literature documents was 700-1300 ppm.

To identify alternatives, understanding ZnO’s mechanism is essential. Dietary supplementation with high levels of ZnO has been demonstrated to exert significant modulatory effects on the intestinal microbial composition and diversity in weaned piglets, particularly through enhancing the resilience of gut microbiota homeostasis during the critical postweaning developmental phase (Vahjen et al., 2011). A recent study further indicates that ZnO plays a beneficial role in microbiota transition after weaning, preventing the overgrowth of pathogens such as pathogenic *E. coli* and highlighting key aspects of microbiota maturation that ZnO alternatives should address (Ortiz Sanjuán et al., 2024). For instance, sodium alginate-coated nano ZnO has been demonstrated to reduce microbial alpha diversity, modulate gut microbiota structure, and serve as a feasible substitute for conventional ZnO to mitigate zinc emissions (Xiao et al., 2023). Moreover, elucidating the molecular mechanisms through which zinc regulates intestinal barrier function in piglets could be critical for evaluating the efficacy of potential ZnO alternatives in weaned piglet nutrition. Studies show that Zn strengthens the porcine jejunal epithelial barrier by reversibly tightening the paracellular route for inorganic ions and small solutes, while acute or long-lasting Zn effects on transcellular transport were not detected (Zakrzewski et al., 2017). Therefore, zinc sources with enhanced bioavailability demonstrate a dose-dependent improvement in porcine intestinal health parameters, suggesting their therapeutic potential as functional alternatives to pharmacological ZnO supplementation.

ZnL is an organic-acid-trace-element chelate with high solubility and bioavailability. Dietary supplementation with organic zinc, particularly 100 ppm ZnL has demonstrated significant efficacy in enhancing growth performance and improving gut barrier function in weaned piglets (Diao et al., 2021). Pre-administration of ZnL at 80 ppm has been reported to suppress intestinal oxidative stress in piglets by enhancing antioxidant capacity and regulating gut microbiota (Tang et al., 2024). However, limited research exists on whether ZnL can effectively replace high-dose ZnO in weaned piglet diets. Based on optimal dosage studies of other alternative zinc sources, we hypothesized that the administration of ZnL could improve the composition of gut microbiota, enhance antioxidant capacity, promote gut health, and reduce the rate of diarrhea, as well as generate comparable effects to those of high-dose ZnO. Therefore, the level of ZnL at 600–800 ppm was used to compare the outcomes of high-dose ZnO and ZnL on the growth performance, serum biochemical parameters, intestinal morphology, and gut microbiota composition in weaned piglets in this study.

## Materials and methods

### Ethics approval

The animal study was reviewed by the Animal Care and Use Committee of Leshan Normal University (Certification No. 4151010649), China, and conducted in accordance with the approved protocol (No. LAC2024001). Written informed consent was obtained from the owners for their animals’ participation in this study.

### Animals and experimental design

A total of 128 healthy 25-day-old piglets (Duroc × Landrace × Yorkshire), with average body weight of 7.31 ± 0.25 kg, were selected for an 18-day experiment. The piglets were randomly allocated into four groups: (1) negative control group (CON-), a basal diet without supplemental ZnO; (2) positive control group (CON+), basal diet + 1,500 ppm zinc with ZnO; (3) experimental groups 1 (TRE1), basal diet + 600 ppm zinc with ZnL (99%; Hunan Debang Biotechnology Co., Ltd.); (4) experimental groups 2 (TRE2), basal diet + 800 ppm zinc with ZnL. Each group had four replicates, with 8 piglets per replicate (half barrows and half gilts). The basal diet was formulated according to the National Nutrient Requirements of Swine Council (2012), as shown in Table 1. The addition level of zinc lactate is determined by referring to other organic zinc source additives (Peng et al., 2024). No antibiotics were used in any diet, and the comprehensive acidifier was removed in TRE1 and TRE2 groups for cost considerations. Samples of the feed were assessed to determine the contents of zinc via atomic absorption spectrometry (Chinese National Standard, GB/T 13885–2017). The whole trial lasted for 18 days. The experimental piglets had access to feed (in a mash form) and water *ad libitum* during the experiment. All piglets were fed in 1.7 × 2.1 m^2^ pens with plastic slatted floors in a room maintained at a temperature of 26–28°C throughout the experimental period.

**Table 1:** Composition and nutritional level of basic diets (As fed-basis).

Ingredients	Content (%)	Composition	Nutrient level^c^
Corn	26.10	Digestible energy DE (MJ/kg)	14.80
Puffed rice	15.00	Crude protein CP (%)	17.85
Puffed soybeans	11.50	Crude fat EE (%)	8.61
Soybean protein hydrolysate	13.00	Lysine Lys (%)	1.45
High-protein whey powder	10.00	Methionine Met (%)	0.54
Low-protein whey powder	10.00	Threonine Thr (%)	0.94
Glucose	2.50	Tryptophan Trp (%)	0.27
Sucrose	2.50	Znic (ppm)^d^	131
Yeast hydrolysate	2.00		
Soybean oil	2.50		
Calcium dihydrogen phosphate	1.20		
L-lysine hydrochloride	0.60		
Calcium lactate	0.50		
Comprehensive acidifier^b^	0.40		
Zeolite powder	0.20		
L-threonine	0.35		
Sodium chloride	0.30		
DL-methionine	0.25		
Choline chloride	0.10		
Premix^a^	1.00		
Total	100.00		

a The premix provides per kg of feed: VitA 13,125 IU, VitD3 3,125 IU, VitE 111 IU, VitK3 6.3 mg, VitB1 3.3 mg, VitB2 13.2 mg, VitB6 7.7 mg, VitB12 0.063 mg, niacin 64.0 mg, pantothenic acid 30.6 mg, folic acid 2.0 mg, biotin 0.3 mg, Fe 215 mg, Cu 103 mg, Zn 110 mg, Mn 32 mg, Se 0.47 mg, I 0.2 mg.

b Acidifier: A compound acidifier composed of lactic acid, citric acid, phosphoric acid, etc., with a total acid content of 80%.

c The nutritional levels are calculated values.

d Analyzed zinc content of experiment diets were as follows: 131 ppm (CON-), 1637 ppm (CON+), 732 ppm (TRE1), 930 ppm (TRE2).

### Growth performance, diarrhea rate, and sensory score

On days 1 and 18 of the experiment, the initial body weight (IBW) and final body weight (FBW) of the piglets were measured after a 12 h fast to calculate average daily gain (ADG). The feed consumption from each pen was recorded to evaluate average daily feed intake (ADFI), and feed intake to gain ratio (FCR). Fecal scores and the diarrhea rate were evaluated as previously described by Dai et al. (2024b). The scoring criteria (1–5 points) are shown in Table 2 according to Zhang et al. (2021). The number of diarrhea piglets (scores of 3 points and above was considered diarrhea) was recorded daily for each replicate. Diarrhea rates and average fecal scores were calculated for the first week, second week, and the entire experimental period. No diarrhea was observed after the first 2 weeks, so no further diarrhea-related data were collected.

**Table 2:** Scoring standards for feces of weaned piglets^a^.

Score	Scoring criteria	Assess
1	Stool is hard or granular	Uncommon
2	Normal feces, soft and shaped	No diarrhea
3	Loose feces, partially formed	Mild diarrhea
4	Semi liquid feces, fecal water not separated	Moderate diarrhea
5	Water sample feces, fecal water separation	Severe diarrhea

a Scoring standards for feces were according to Zhang et al. (2021).

On days 7 and 14 of the experiment, all piglets were scored using the fur scoring standard in Table 3 (Guo et al., 2005), and the average skin and fur score was calculated. Average skin and fur score = (skin redness score + fur brightness score + dishevelled fur score)/3. The assessment of fur score and faecal score was carried out by two experimental staff who were trained together according to the reported method of Lange et al. (2020), one being the assessor, the other one an active observer, always ready to justify the given score.

**Table 3:** Scoring standards for skin and fur of weaned piglets^a^.

Score	Degree of skin redness	Fur brightness	Degree of dishevelled fur
1	Pale	Matte	Obviously messy
2	slightly red	Faint gloss	Slightly messy
3	Obvious ruddy	Clear gloss	Obviously compliant

a Scoring standards for skin and fur were according to Guo et al. (2005).

### Sample collection

On day 18, one piglet per pen (2 barrows and 2 gilts per group), selected based on the average body weight, was sampled for blood collection from the precaval vein. The blood samples were centrifuged at 3,000 × *g* for 10 min at −4°C to obtain serum, and stored at −20°C for further analysis.

At the end of the experiment, piglets that had provided blood samples were anesthetized and euthanized, and the intestine was removed for sample collection. The middle parts of the duodenum, jejunum, and ileum were excised and cut down the 2 cm gut segment, rinsed with pre-cooled PBS, blotted dry with filter paper, flash-frozen in liquid nitrogen, and stored at −80°C to detect gene expression of barrier function. Cecal contents (approximately 5 g per sample) were also collected, flash-frozen, and stored at −80°C for microbial detection. Additionally, approximately 0.5 cm segments of the duodenum, jejunum, and ileum were fixed in 4% formaldehyde for histological analysis.

### Serum indexes

The activities of antioxidant enzymes-including glutathione peroxidase (GPx), superoxide dismutase (SOD), copper-zinc superoxide dismutase (Cu/Zn-SOD), and the content of malondialdehyde (MDA) in serum were measured by series kits according to the protocols (Nanjing Jiancheng Bioengineering Institute, Nanjing, China). The product numbers of the commercial kit were A005-1-2, A001-3-2, A001-2-2 and A003-1-2, respectively.

### Analysis of intestinal morphology

The fixed segments (approximately 1 cm) of the duodenum, jejunum and ileum were dehydrated, trimmed, embedded, and sliced into 5 μm (Leica-2016 rotary tissue slicer, Germany). The sections were stained with haematoxylin and eosin, and then sealed. Three images of intestinal morphology (40 × magnification) were collected by BA210 Digital three-lens camera system (SPEED FAIR CO., LTD.). The villus height, villus width and crypt depth were measured by image analysis software. Approximately 10 groups of data were measured from each slice sample, and the average value was used as one measurement data. The ratio of villus height/crypt depth (VH/CD) was calculated.

### RT-qPCR analysis for gene expression

The mRNA expression levels of tight junction protein in intestine were evaluated as previously described by Dai et al. (2024b). Approximately 100 mg of duodenal, jejunal, and ileal tissue samples were homogenized in RNA-free 1.5 mL tubes. Total RNA was extracted using a column-based TRIzol kit (TaKaRa Biotechnology, China) according to the manufacturer’s instructions. The integrity of RNA was assessed by agarose gel electrophoresis, and concentration was measured using a spectrophotometer. Reverse transcription cDNA synthesis was conducted by using oligo (dT)20 and Superscript II reverse transcriptase (Invitrogen). The primers for tight junction protein Claudin-1(*CLDN-1*), Occludin (*OCLN*), and Zonula occludens 1 (*ZO-1*), and internal reference gene (*β*-actin) were designed as shown in Table 4. A real-time PCR kit (SYBR green, TaKaRa) was performed to quantify mRNA expression levels. The PCR protocol was as follows: 95°C for 1 min; 40 cycles at 95°C for 10 s, 60°C for 15 s, and 72°C for 15 s; and a final extension at 72°C for 4 min. Each sample was run in duplicate on Bole fluorescent quantitative PCR instrument (QTOWER, Analytik Jena AG, German). The relative mRNA expression levels of the target gene in comparison with the reference gene were calculated by the 2^−ΔΔCt^ method (Livak and Schmittgen, 2001).

**Table 4:** Primer sequences used in real-time fluorescence quantitative RT-PCR.

Gene	Gene bank NO.	Primers sequence	Amplification length (bp)
*β-actin*	XM-021086047.1	F:5’-TCTGGCACCACACCTTCT-3’	114
		R:5’-TGATCTGGGTCATCTTCTCA-3’	
*CLDN-1*	NM_001161635.1	F:5’-GGACTAATAGCCATCTTGT-3’	88
		R:5’-CAGCCATCCGCATCTTCT-3’	
*OCLN*	NM_001163647.1	F:5’-ATGCTTTCTCAGCCAGCGTA-3’	176
		R:5’-AAGGTTCCATAGCTCGGTC-3’	
*ZO-1*	XM003353439.1	F:5’-GAGGATGGTCACACCGTGGT-3’	169
		R:5’-GGAGGATGCTGTTGTCTCGG-3’	

### Microbial analysis based on 16S rRNA sequencing

Total genomic DNA was extracted from the cecal digesta samples using a DNA extraction kit (Omega Bio-tek, Norcross, GA, USA). The quality of the isolated DNA was assessed by 1% agarose gel electrophoresis, and concentration and purity were determined using a NanoDrop2000 (Thermo Scientific Inc., USA). The V3-V4 gene region of the 16S rRNA was amplified using the primers 338F (5’-ACTCCTACGGGAGGCAGCAG-3′) and 806R (5’-GGACTACHVGGGTWTCTAAT-3′) as described by Liu et al. (2016). The 16S rRNA gene were sequenced on the Illumina Miseq PE300 platform by Shanghai Majorbio Biomedical Technology Co., Ltd. Related analysis was performed using the online platform of Majorbio Cloud Platform (^1^) as previously described by Dai et al. (2024a). The raw reads have been deposited into NCBI with project NO. PRJNA1258601.

Fastp software (https://github.com/OpenGene/fastp, version 0.19.6) was used for quality control of double-ended original sequencing sequences (Chen et al., 2018). FLASH software (http://www.cbcb.umd.edu/software/flash, version 1.2.11) was applied for stitching (Magoc and Salzberg, 2011). Operational taxonomic units (OTUs) were clustered at 97% indentity using UPARSE (http://drive5.com/uparse/, version 7.1), and chimeras were removed. Sequences were rarefied to 46,592 per sample to standardize sequencing depth. Taxonomic annotation was performed using the RDP classifier against the Silva 16S rRNA database (Release 138), with a confidence threshold of 70%.

### Statistical analysis

Data were analyzed by a *t*-test or one-way ANOVA using SPSS 23.0 statistical software (IBM company, USA). The statistical model included dietary treatment (ZnO or ZnL), and LSD method was used for multiple comparisons. Growth performance, diarrhea, serum indexes, intestinal morphology, and gene expression were statistically analyzed. For 16S rRNA sequencing analysis, ɑ diversity (Ace, Chao, Sobs, Shannon, and Simpson) was calculated using mothur^2^ (Schloss et al., 2009). β diversity was assessed using PCoA based on Bray-Curtis distances of OTU relative abundance, and the difference among groups was tested by the analysis of similarities (ANOSIM). The differences between groups at the phylum and genus levels were analyzed by the Wilcoxon rank-sum test. Spearman correlation analysis was applied to evaluate the correlations between top 10 genera and piglets’ performance. The results were reported as mean ± standard error, *p* < 0.05 indicated significant differences, and *p* < 0.10 indicated a difference trend.

## Results

### Effects on growth performance and diarrhea incidence in piglets

Growth performance parameters of piglets are summarized in Table 5. The ADFI in the CON+ group was significantly higher than TRE1 (*p* < 0.05), and tended to be higher than CON- (*p* = 0.102), whereas no significant difference was observed between TRE2 and CON+ (*p* > 0.05). No statistically significant difference were detected among groups for FBW, ADG, and FCR (*p* > 0.05). Notably, while the CON+ group showed numerically higher ADG and improved FCR compared to CON-, TRE2 displayed comparable performance to CON+ with 11.36 and 11.93% enhancements in ADG and FCR, respectively, relative to CON-. Furthermore, there was no significant difference in the overall ANOVA results of ADFI, but there was a significant difference compared with the *post hoc* test of TRE1 and CON+, which might be caused by insufficient efficacy.

**Table 5:** Effects of ZnL on the growth performance of piglets.

Items	CON−	CON+	TRE1	TRE2	SEM	*P-*value
IBW (Kg)	7.29 ± 0.11	7.40 ± 0.25	7.27 ± 0.18	7.26 ± 0.03	0.07	0.927
FBW (Kg)	12.72 ± 0.41	13.43 ± 0.39	12.83 ± 0.44	13.31 ± 0.55	0.21	0.631
ADFI (g/d)	467.67 ± 5.24^ab^	477.00 ± 0.58^b^	445.00 ± 7.23^a^	456.44 ± 14.84^ab^	5.17	0.128
ADG (g/d)	301.67 ± 17.29	335.00 ± 12.50	309.00 ± 15.31	335.95 ± 29.30	9.57	0.525
FCR	1.56 ± 0.09	1.43 ± 0.05	1.45 ± 0.08	1.38 ± 0.08	0.04	0.430

The shoulder labels of the peer data without letters or with the same letters indicate no significant difference (*P* > 0.05), while different letters indicate significant difference (*P* < 0.05).

Diarrhea incidence and fecal consistency (FC) scores of the piglets are detailed in Table 6. Compared with the CON- group, the CON+ group exhibited significantly reduced the diarrhea rate in 2^nd^ week and lower average diarrhea rate (*p* < 0.05). Similarly, the TRE2 group showed a trend toward reduced the diarrhea rate in 2^nd^ week (*p* < 0.10) and significantly decreased average diarrhea rate compared to CON- (*p* < 0.05), with no statistically significant difference observed between CON+ and TRE2 (*p* > 0.05). Regarding fecal consistency, CON+ demonstrated significantly lower FC scores in 2^nd^ week and improved average FC scores relative to CON- (*p* < 0.05). Although neither TRE1 nor TRE2 achieved statistical significance in FC scores compared to CON+ (*p* > 0.05), both treatment groups exhibited numerically reduced values versus CON- (*p* > 0.05), suggesting a potential dose-responsive ameliorative pattern.

**Table 6:** Effects of ZnL on diarrhea rate of piglets.

Items	CON−	CON+	TRE1	TRE2	SEM	*P*-value
Diarrhea rate in 1^st^ week(%)	19.29 ± 4.42	9.29 ± 2.14	13.57 ± 5.13	8.57 ± 3.09	2.05	0.237
Diarrhea rate in 2^ed^ week(%)	17.14 ± 3.69^b^	0.72 ± 0.72^a^	10.72 ± 4.27^b^	7.86 ± 2.44^ab^	2.06	0.020
Average diarrhea rate in 2 weeks (%)	18.22 ± 1.79^b^	5.00 ± 1.24^a^	12.14 ± 4.61^ab^	8.21 ± 1.07^a^	1.72	0.021
Fecal score in 1^st^ week	2.19 ± 0.10	2.06 ± 0.03	2.10 ± 0.06	2.20 ± 0.09	0.04	0.490
Fecal score in 2^ed^ week	2.29 ± 0.07^b^	2.02 ± 0.01^a^	2.17 ± 0.04^ab^	2.14 ± 0.10^ab^	0.04	0.079
Average fecal score in 2 weeks	2.24 ± 0.06^b^	2.04 ± 0.01^a^	2.13 ± 0.05^ab^	2.16 ± 0.06^ab^	0.03	0.085

The shoulder labels of the peer data without letters or with the same letters indicate no significant difference (*P* > 0.05), while different letters indicate significant difference (*P* < 0.05).

### Effects on skin and fur sensory indicators of piglets

The sensory scores of skin and fur in piglets are detailed in Table 7. On day 7, the CON+ group demonstrated reduced skin redness score compared to CON- (*p* = 0.102), whereas TRE1 exhibited significantly elevated skin redness scores versus CON+ (*p* < 0.05), and TRE2 showed a tendency towards a higher score than CON+ (*p* < 0.10). Regarding the fur brightness score, CON+ showed a significantly lower value than CON- (*p* < 0.05), while both TRE1 (*p* = 0.108) and TRE2 (*p* < 0.10) tended to have higher scores than CON+. Furthermore, CON+ tended to have lower average skin and fur scores than CON- (*p* < 0.10), and TRE2 showed a tendency towards a higher score than CON+ (*p* < 0.10). By day 14, there was no significant differences in the sensory scores of skin and fur among these groups (*p* > 0.05).

**Table 7:** Effects of ZnL on the sensory score of skin and fur of piglets.

Items	CON−	CON+	TRE1	TRE2	SEM	P-value
7d						
Skin redness score	2.85 ± 0.10^ab^	2.50 ± 0.24^a^	2.95 ± 0.05^b^	2.90 ± 0.10^ab^	0.08	0.150
Fur brightness score	2.80 ± 0.14^b^	2.45 ± 0.13^a^	2.70 ± 0.06^ab^	2.74 ± 0.05^ab^	0.06	0.132
Dishevelled fur score	2.65 ± 0.22	2.55 ± 0.10	2.50 ± 0.10	2.74 ± 0.05	0.06	0.607
average skin and fur score	2.77 ± 0.13	2.50 ± 0.14	2.72 ± 0.04	2.79 ± 0.06	0.05	0.227
14d						
Skin redness score	2.95 ± 0.05	3.00 ± 0.00	3.00 ± 0.00	3.00 ± 0.00	0.01	0.426
Fur brightness score	2.85 ± 0.10	2.95 ± 0.05	3.00 ± 0.00	3.00 ± 0.00	0.03	0.217
Dishevelled fur score	2.75 ± 0.10	2.75 ± 0.13	2.90 ± 0.10	2.84 ± 0.06	0.05	0.650
average skin and fur score	2.85 ± 0.06	2.90 ± 0.04	2.97 ± 0.03	2.95 ± 0.02	0.02	0.229

The shoulder labels of the peer data without letters or with the same letters indicate no significant difference (*P* > 0.05), while different letters indicate significant difference (*P* < 0.05).

### Effects on serum indexes of piglets

The serum indexes of piglets are detailed in Table 8. Compared with the CON- group, both TRE1 and TRE2 demonstrated marked elevation serum GPx activity (*p* < 0.05). The serum SOD activity was significantly enhanced in CON+ and TRE2 groups compared to CON- (*p* < 0.05). Similarly, the serum Cu/Zn-SOD activity in the CON+, TRE1, and TRE2 groups was significantly elevated compared to CON- (*p* < 0.05). Notably, serum MDA concentrations in the CON+ group displayed significantly lower levels than CON-, TRE1, and TRE2 (*p* < 0.05).

**Table 8:** Effects of ZnL on serum indexes of piglets.

Items	CON−	CON+	TRE1	TRE2	SEM	*P*-value
GPx (U/mL)	428.18 ± 23.30^a^	456.14 ± 17.15^ab^	486.82 ± 4.24^b^	499.77 ± 8.86^b^	9.91	0.026
SOD (U/mL)	162.69 ± 2.29^a^	220.20 ± 7.72^b^	196.36 ± 20.61^ab^	201.97 ± 7.24^b^	7.48	0.029
Cu/Zn-SOD (U/mL)	72.01 ± 2.42^a^	102.33 ± 4.32^b^	92.23 ± 2.42^b^	99.81 ± 5.60^b^	3.54	0.001
MDA (μmol/L)	9.16 ± 0.93^b^	6.58 ± 0.27^a^	9.10 ± 0.30^b^	8.39 ± 0.55^b^	0.37	0.027

The shoulder labels of the peer data without letters or with the same letters indicate no significant difference (*P* > 0.05), while different letters indicate significant difference (*P* < 0.05).

### Effects on intestinal histology of piglets

Intestinal histology of piglets are presented in Table 9. ZnL supplementation improved development of intestinal villus height. TRE2 and CON+ exhibited greater villus height in the duodenum compared to CON- (*p* < 0.05), and both TRE1 and TRE2 showed significantly higher villus height in the jejunum compared to CON- (*p* < 0.05). However, TRE1 showed lower villus height of the ileum than CON- and TRE2 (*p* < 0.05). TRE1 displayed significantly reduced crypt depth in the duodenum relative to all other groups (*p* < 0.05), and TRE2 showed significantly increased crypt depth in the duodenum relative to all other groups (*p* < 0.05). Notably, there were no significant differences in the VH/CD ratio among groups (*p* > 0.05). The jejunal villus width in the CON+ and TRE1 groups increased significantly compared with CON- (*p* < 0.05), and the ileal villus width in the CON+ group was significantly higher than that in all other groups (*p* < 0.05).

**Table 9:** Effects of ZnL on intestinal morphology of piglets.

Items	CON−	CON+	TRE1	TRE2	SEM	*P*-value
Villus height (μm)						
Duodenum	413.85 ± 16.92^a^	523.48 ± 38.94^b^	437.93 ± 18.25^a^	538.14 ± 24.82^b^	15.23	0.003
Jejunum	300.02 ± 25.41^a^	339.19 ± 17.38^ab^	364.46 ± 29.23^b^	376.06 ± 14.48^b^	11.74	0.099
Ileum	388.12 ± 11.27^b^	360.98 ± 29.95^ab^	322.83 ± 19.98^a^	413.07 ± 23.19^b^	11.91	0.041
Crypt depth (μm)						
Duodenum	387.58 ± 28.04^b^	386.57 ± 18.02^b^	325.68 ± 14.63^a^	472.24 ± 14.58^c^	12.59	<0.001
Jejunum	270.66 ± 17.31	306.88 ± 12.53	313.14 ± 23.56	321.02 ± 37.80	12.26	0.495
Ileum	239.62 ± 24.40	270.29 ± 34.97	241.64 ± 7.91	261.31 ± 19.19	11.58	0.753
VH/CD ratio						
Duodenum	1.13 ± 0.11	1.41 ± 0.15	1.38 ± 0.09	1.15 ± 0.07	0.06	0.178
Jejunum	1.17 ± 0.15	1.13 ± 0.09	1.29 ± 0.19	1.44 ± 0.26	0.09	0.653
Ileum	1.84 ± 0.25	1.71 ± 0.39	1.37 ± 0.12	1.65 ± 0.13	0.12	0.579
Villus width(μm)						
Duodenum	137.10 ± 13.08	190.16 ± 20.90	164.85 ± 9.69	161.66 ± 13.68	7.77	0.115
Jejunum	98.64 ± 6.71^a^	126.72 ± 10.33^b^	159.40 ± 6.42^c^	112.45 ± 12.67^ab^	5.78	<0.001
Ileum	108.29 ± 6.80^a^	148.53 ± 7.26^b^	116.23 ± 12.01^a^	99.55 ± 9.17^a^	5.26	0.003

The shoulder labels of the peer data without letters or with the same letters indicate no significant difference (*P* > 0.05), while different letters indicate significant difference (*P* < 0.05).

### Effects on mRNA expressions of tight junction proteins of piglets

The mRNA expressions of tight junction proteins in the intestines of piglets are detailed in Table 10. TRE2 showed significantly higher mRNA expression of *CLDN-1* in the duodenum and ileum of piglets than all other groups (*p* < 0.05). Additionally, TRE2 group showed higher mRNA expression of *CLDN-1* in the jejunum than CON+ (*p* < 0.05). However, there were no significant differences in the mRNA expressions of *OCLN* and *ZO-1* among groups (*p* > 0.05).

**Table 10:** Effects of ZnL on mRNA expressions of tight junction proteins of piglets.

Items	CON−	CON+	TRE1	TRE2	SEM	*P*-value
CLDN-1						
Duodenum	1.04 ± 0.15^a^	0.95 ± 0.47^a^	1.76 ± 0.99^a^	5.68 ± 1.68^b^	0.67	0.018
Jejunum	1.02 ± 0.0.18^ab^	0.43 ± 0.04^a^	1.31 ± 0.35^ab^	1.84 ± 0.50^b^	0.23	0.186
Ileum	1.18 ± 0.42^a^	3.66 ± 0.57^a^	27.78 ± 15.19^a^	88.22 ± 30.18^b^	12.46	0.020
OCLN						
Duodenum	1.03 ± 0.14	3.33 ± 1.52	3.26 ± 2.49	4.42 ± 2.16	0.87	0.618
Jejunum	1.03 ± 0.17	2.86 ± 2.14	2.57 ± 1.14	3.23 ± 0.55	0.55	0.606
Ileum	1.18 ± 0.50	3.29 ± 1.41	3.45 ± 1.50	11.40 ± 8.57	2.35	0.473
ZO-1						
Duodenum	1.06 ± 0.19	3.72 ± 1.74	4.95 ± 3.14	5.66 ± 3.37	1.19	0.584
Jejunum	1.18 ± 0.40	2.03 ± 1.80	1.92 ± 0.54	1.18 ± 0.71	0.71	0.893
Ileum	1.16 ± 0.47	2.61 ± 1.17	5.51 ± 1.84	15.08 ± 11.42	3.14	0.428

The shoulder labels of the peer data without letters or with the same letters indicate no significant difference (*P* > 0.05), while different letters indicate significant difference (*P* < 0.05).

### Effects on microbial α- and β-diversity levels of piglets

**Figure 1:**
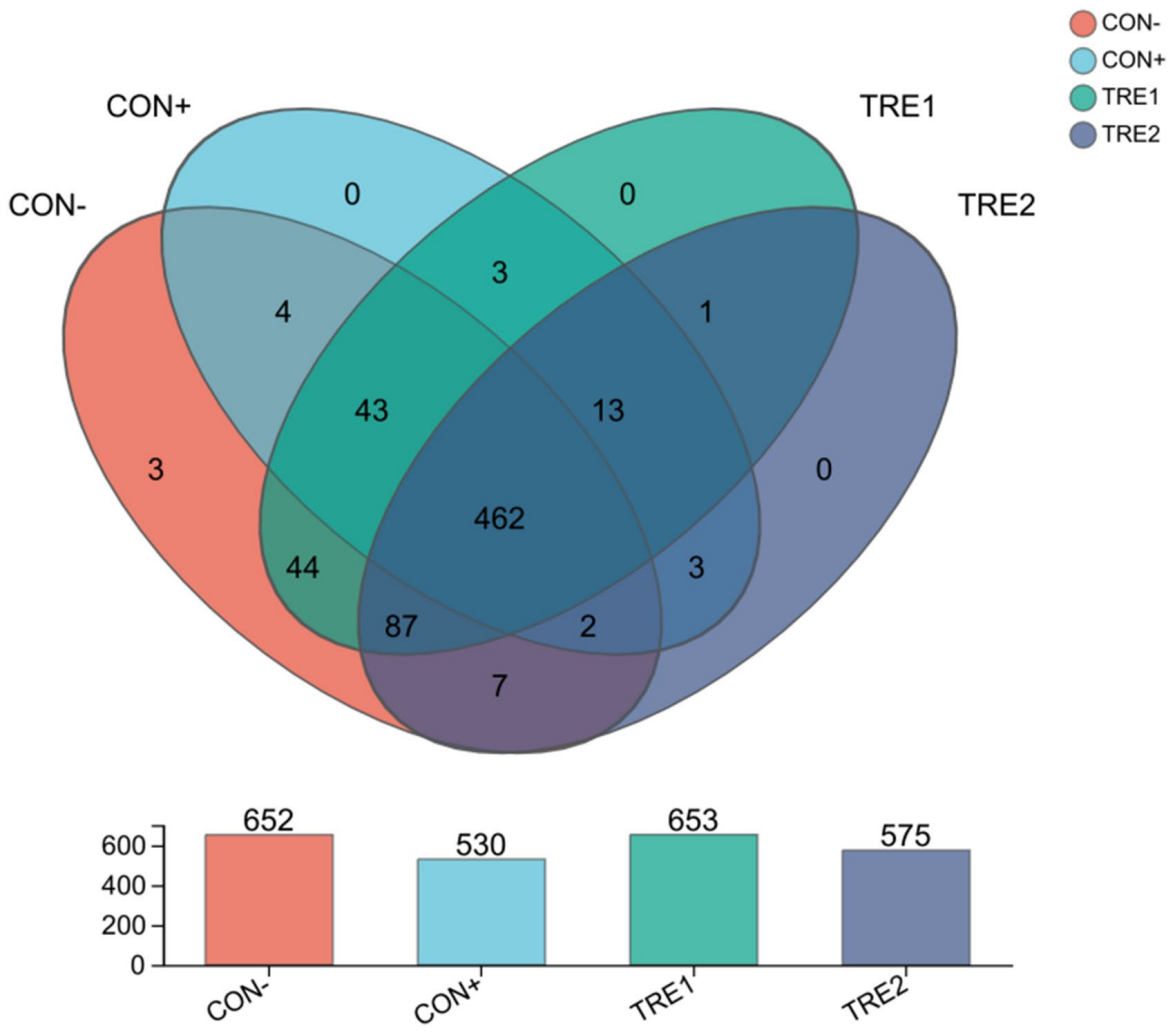
Venn diagram of cecal microbiota OTUs from CON-, CON+, TRE1 and TRE2 piglets.

A total of 1,040,401 sequences were obtained, with an average of 65,025 sequences per sample and an average sequence length of 410 bp. The Good’s coverage for the observed OTUs was 99.90 ± 0.01%. According to the Veen plot (Figure 1), the average numbers of OTUs in CON-, CON+, TRE1 and TRE2 were 652, 530, 653, and 575, respectively. Compared with the CON- group, CON+, TRE1 and TRE2 had 19, 17 and 17 unique OTUs, respectively. The microbial ɑ-diversity indexes are presented in Table 11. Dietary supplementation with ZnL had a significant effect on the richness indexes (Ace, Chao, and Sobs) and diversity indexes (Shannon and Simpson) of cecal digesta microbiota (*p* < 0.05). The Ace, Chao, and Sobs indexes in CON+ and TRE2 were significantly lower than those in CON- and TRE1 (*p* < 0.05). The Shannon index in the CON+, TRE1, and TRE2 groups was significantly lower than that in CON- (*p* < 0.05), while the Simpson index in the CON+ and TRE2 groups was significantly higher than that in CON- (*p* < 0.05). TRE1 showed a tendency towards a higher Simpson index compared to CON- (*p* < 0.10).

**Table 11:** Effects of ZnL on the microbial α-diversity indexes of piglets.

Items	CON−	CON+	TRE1	TRE2	SEM	*P*-value
Ace	535.99 ± 23.89^b^	344.61 ± 29.96^a^	480.71 ± 36.61^b^	357.91 ± 37.10^a^	25.44	0.003
Chao	540.73 ± 25.99^b^	342.20 ± 28.28^a^	494.12 ± 37.68^b^	358.57 ± 35.74^a^	26.34	0.002
Sobs	511.75 ± 25.27^b^	317.50 ± 29.18^a^	448.25 ± 36.27^b^	335.00 ± 34.97^a^	25.13	0.003
Shannon	4.35 ± 0.16^b^	3.28 ± 0.04^a^	3.76 ± 0.23^a^	3.56 ± 0.21^a^	0.13	0.006
Simpson	0.034 ± 0.004^a^	0.093 ± 0.008^b^	0.067 ± 0.013^ab^	0.068 ± 0.014^b^	0.007	0.018

The shoulder labels of the peer data without letters or with the same letters indicate no significant difference (p > 0.05), while different letters indicate significant difference (*P* < 0.05).

**Figure 2:**
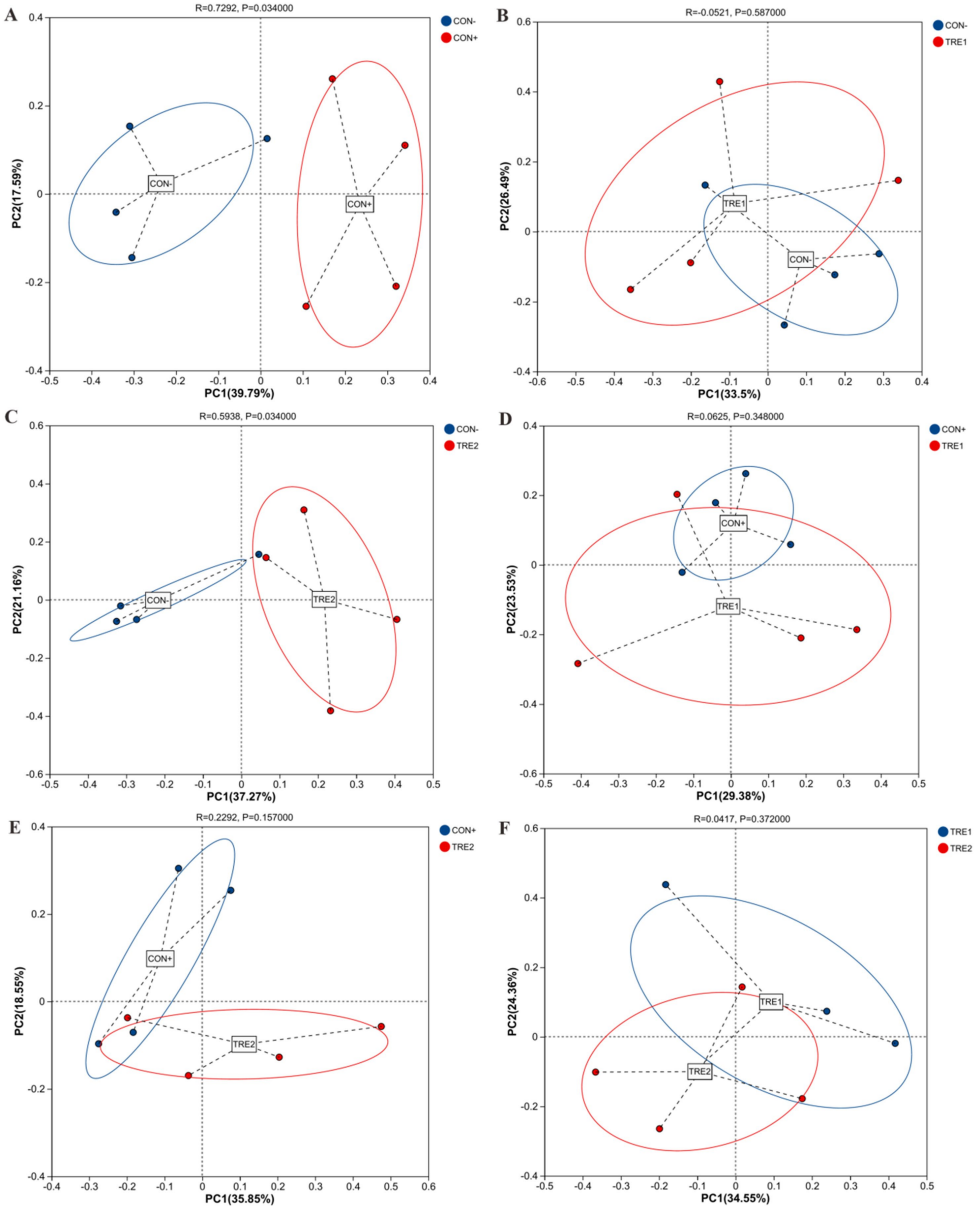
Beta-diversity analysis among experimental groups. Principal coordinates analysis (PCoA) for piglets between CON- and CON+ **(A)**, CON- and TRE1 **(B)**, CON- and TRE2 **(C)**, CON+ and TRE1 **(D)**, CON+ and TRE2 **(E)**, TRE1 and TRE2 **(F)**.

To investigate the effects of ZnL supplementation on the β-diversity, principal coordinate analysis (PCoA) was conducted based on the Bray-Curtis distances of OTU relative abundance in cecal digesta microbiota. As shown in Figure 2, the cecal microbiota of CON+ was distinct from CON- (*R* = 0.729, *p* < 0.05), and TRE2 was also separate from CON- (*R* = 0.594, *p* < 0.05). However, there were no significant separations between the cecal microbiota of CON- and TRE1, CON+ and TRE1, CON+ and TRE2, or TRE1 and TRE2 (*p* > 0.05).

### Effects on microbiota composition of piglets at phyla or genera level

**Figure 3:**
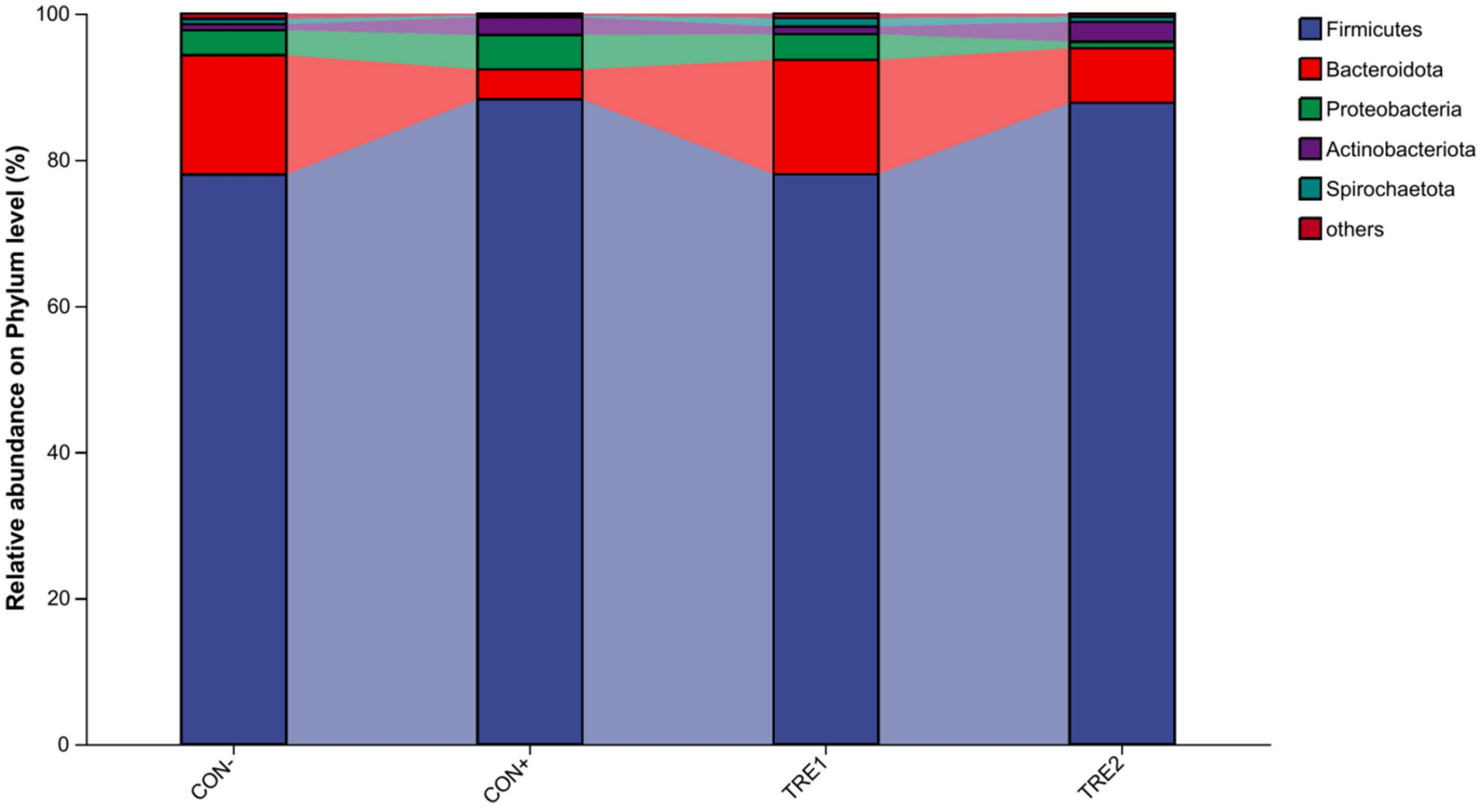
Chyme microbiota composition in piglets of different groups at the phylum level.

The relative abundance of cecal chyme microbiota at the phylum level in different experimental groups is shown in Figure 3. The top 5 dominant phyla were *Firmicutes*, *Bacteroidota*, *Proteobacteria*, *Actinobacteriota,* and *Spirochaetota*. Compared with the CON- group, the proportion of *Firmicutes* in CON+ and TRE2 increased by 10.3 and 9.81%, respectively, while the proportion of *Bacteroidota* decreased by 12.25 and 8.86%, respectively.

**Figure 4:**
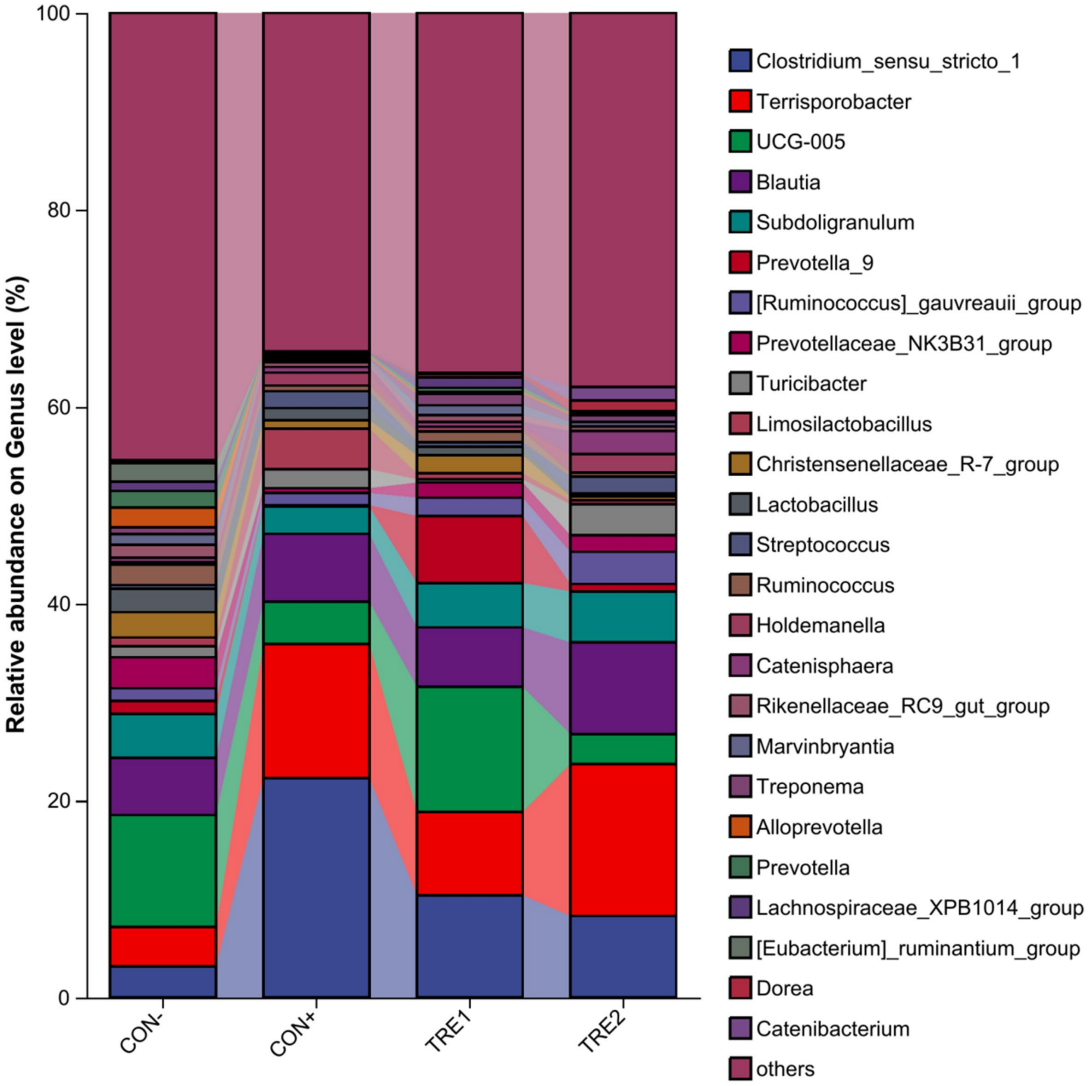
Chyme microbiota composition in piglets of different groups at the genus level.

The composition of cecal chyme microbiota at the genus level in different experimental groups is presented in Figure 4. There were 25 dominant genera across all groups, including *Clostridium_sensu_stricto_1*, *Terrisporobacter*, *UCG-005*, *Blautia*, *Subdoligranulum,* etc. The proportions of dominant genera in the CON-, CON+, TRE1, and TRE2 groups were 54.56, 65.60, 63.45, and 62.00%, respectively. Compared with the CON- group, CON+, TRE1, and TRE2 showed increases in *Clostridium_sensu_stricto_1* (19.13, 7.21, and 5.12%, respectively) and *Terrisporobacter* (9.61, 4.47, and 11.42%, respectively). The *UCG-005* decreased by 7.06 and 8.30% in CON+ and TRE2, respectively, compared to CON-.

**Figure 5:**
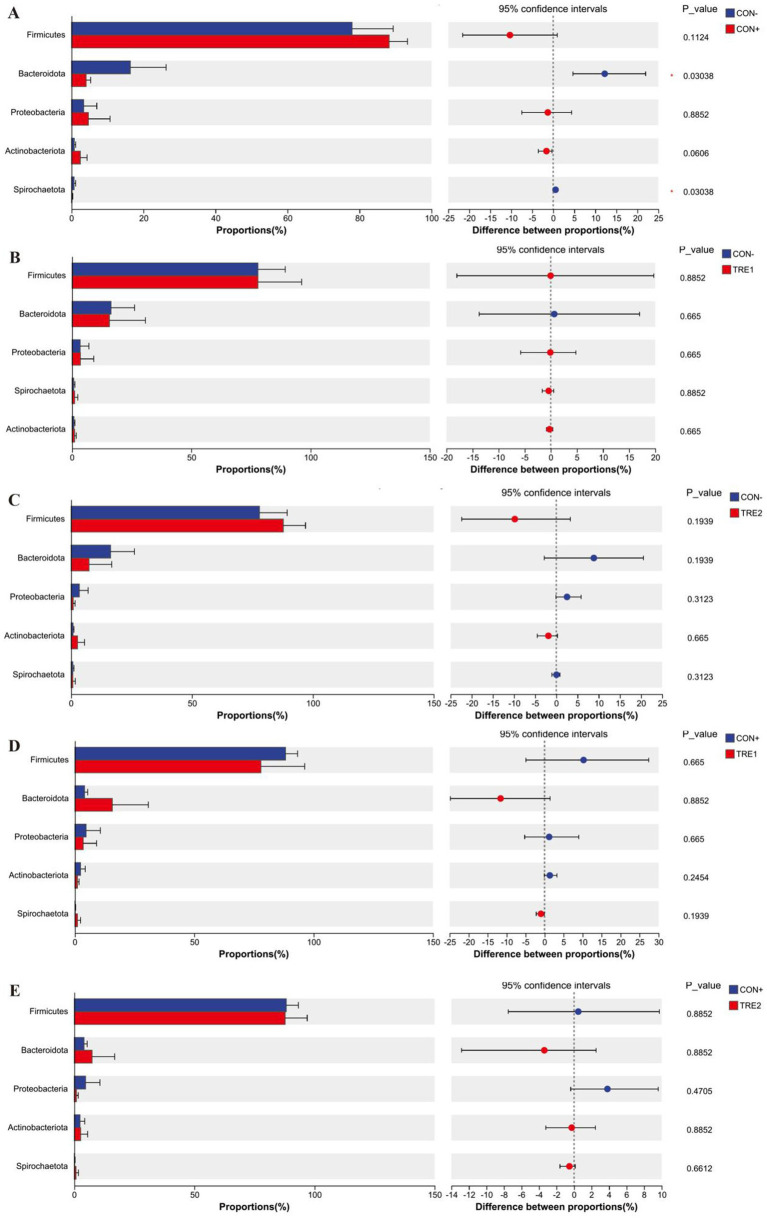
Differences in chyme microbiota at phylum level among experimental groups. Chyme microbiota differed in piglets between the group CON- and CON+ **(A)**, CON- and TRE1 **(B)**, CON- and TRE2 **(C)**, CON+ and TRE1 **(D)**, CON+ and TRE2 **(E)**.

The differences in the top five phyla among the experimental groups are shown in Figure 5. As shown in Figure 5A, compared with the CON- group, CON+ showed significant decreases in the proportion of *Bacteroidota* and *Spirochaetota* (*p* < 0.05). Figures 5B–E indicated that there were no significant differences in the proportions of the main phyla among these groups (*p* > 0.05).

**Figure 6:**
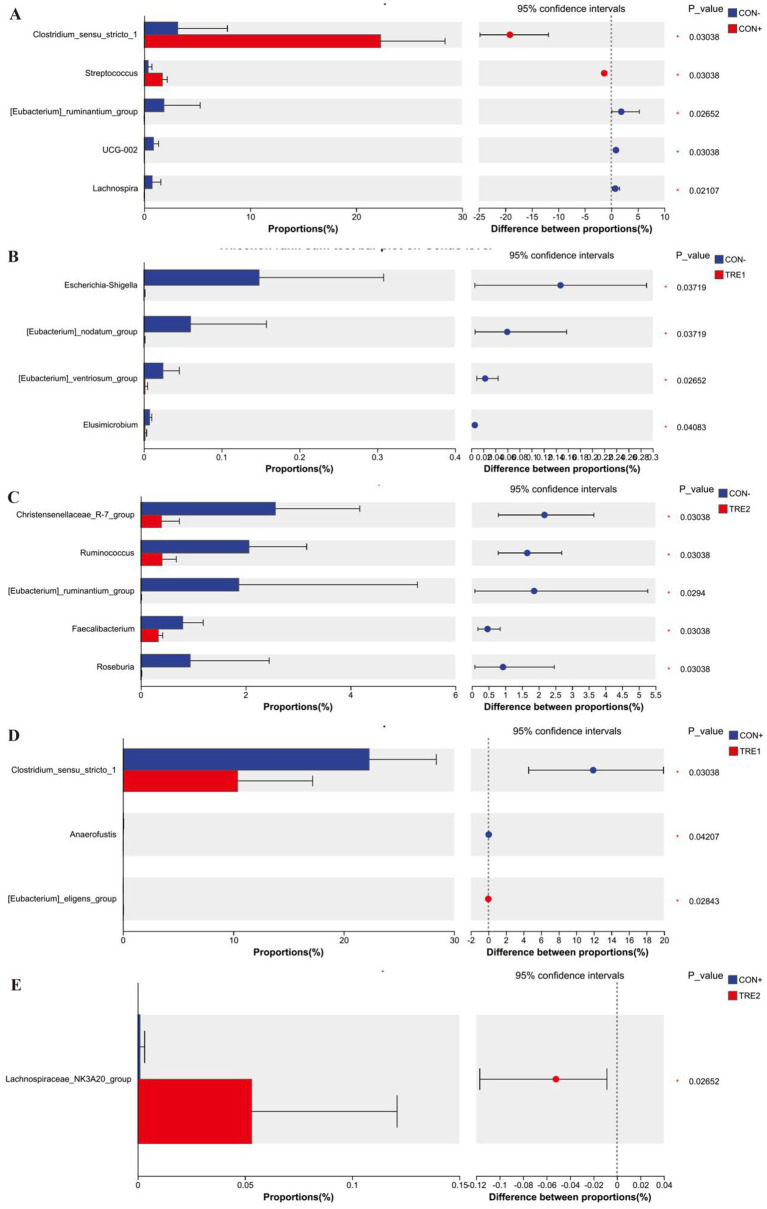
Differences in chyme microbiota at genus level among experimental groups. Chyme microbiota differed in piglets between the group CON- and CON+ **(A)**, CON- and TRE1 **(B)**, CON- and TRE2 **(C)**, CON+ and TRE1 **(D)**, CON+ and TRE2 **(E)**.

The differences in dominant genera among the experimental groups are shown in Figure 6. Genera with proportions exceeding 1% were analyzed. As shown in Figure 6A, compared with the CON- group, CON+ showed a significant increase in *Clostridium_sensu_stricto_1* and *Streptococcu*s (*p* < 0.05) and a significant decrease in *[Eubacterium]_ruminantium_group* (*p* < 0.05). Figure 6C showed that, compared with the CON- group, TRE2 showed a significant decrease in *Christensenellaceae_R-7_group*, *Ruminococcus*, and *[Eubacterium]_ruminantium_group* (*p* < 0.05). Figure 6D indicated that, compared with the CON+ group, TRE1 showed a significant decrease in *Clostridium_sensu_stricto_1* (*p* < 0.05). Figure 6E showed that there was no significant difference in the proportions of genera exceeding 1% between TRE1 and CON- (*p* > 0.05), or between TRE2 and CON+ (*p* > 0.05).

**Figure 7:**
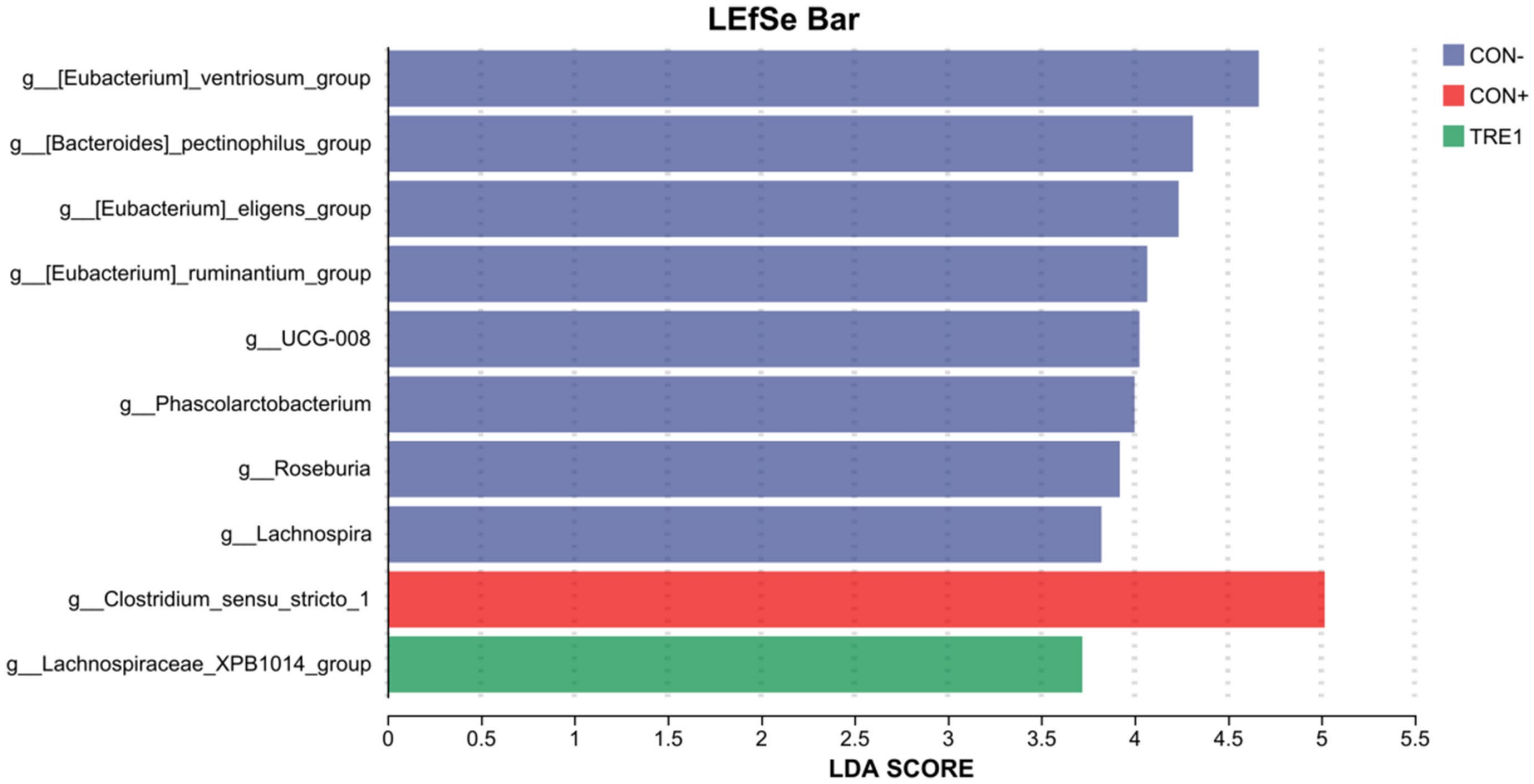
LDA effect size (LEfSe) algorithm was used on genus level OTU tables to determine taxa that best characterize each biological class.

As shown in Figure 7, linear discriminant analysis (LDA) effect size (LEfSe) analysis (LDA threshold = 2) revealed that [Eubacterium]_ventriosum_group, [Bacteroides]_pectinophilus_group, [Eubacterium]_eligens_group, [Eubacterium]_ruminantium_group, UCG-008, Phascolarctobacterium, Roseburia, and Lachnospira were significantly enriched in CON-, while Clostridium_sensu_stricto_1 was significantly enriched in CON+, and Lachnospiraceae_XPB1014_group was significantly enriched in TRE1.

**Figure 8:**
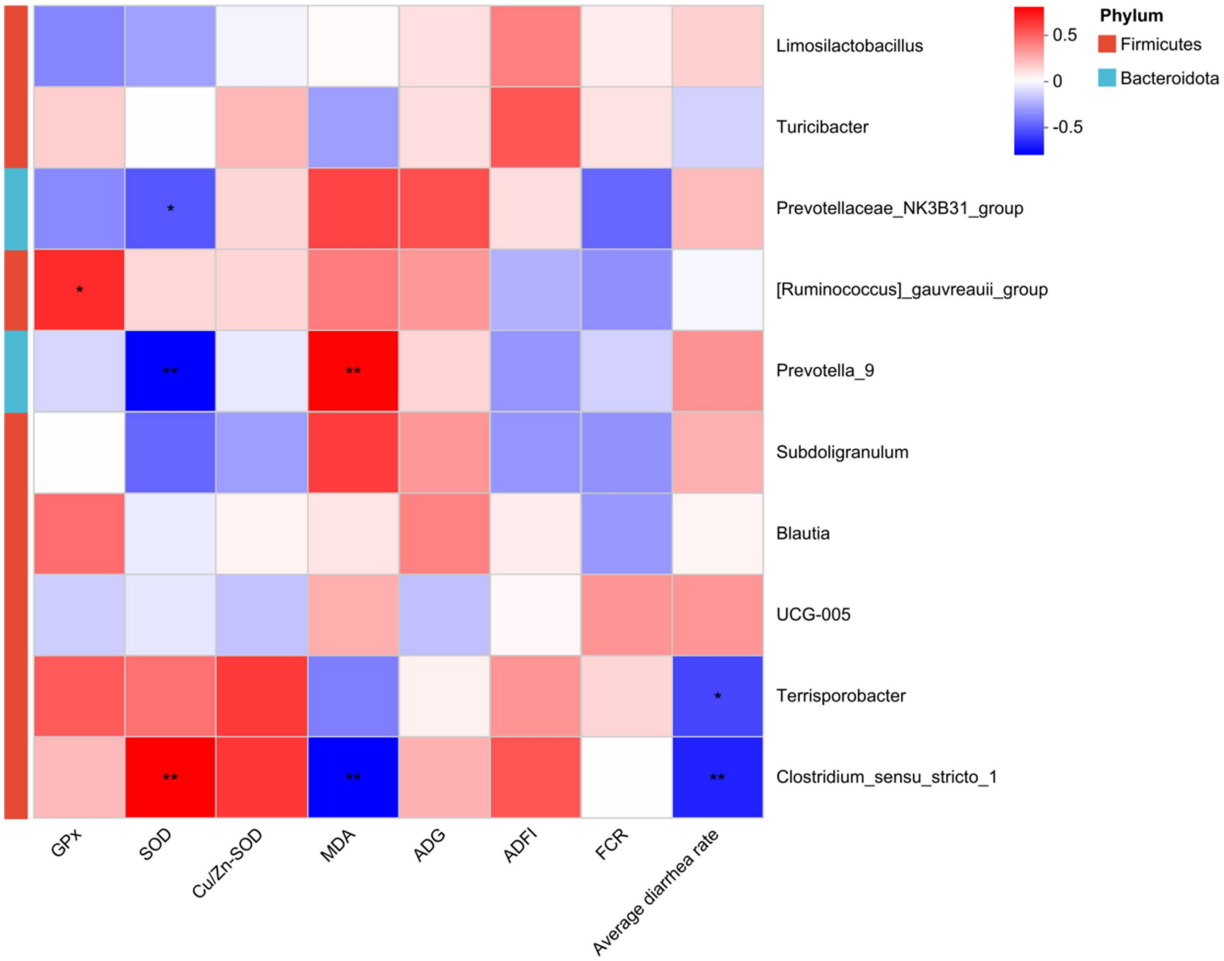
Spearman correlation analysis between differential genera and piglets’ performance. Significant correlations are noted by: *p < 0.05, **p < 0.01.

As shown in Figure 8, spearman correlation analysis revealed that the abundance of *Clostridium_sensu_stricto_1* in the *Firmicutes* phylum (*p* < 0.01) was significantly positively correlated with serum SOD activity, while *Prevotellaceae_NK3B31_group* (*p* < 0.01) and *Prevotella _9* (*p* < 0.05) were significantly negatively correlated with SOD. Meanwhile, the abundance of *[Ruminococcus]_gauvreauii_group* as significantly positively correlated with serum GPx activity (*p* < 0.05). Conversely, the abundance of *Prevotella_9* was significantly positively correlated with serum MDA concentration (*p* < 0.01), and *Clostridium_sensu_stricto_1* was significantly negatively correlated with MDA (*p* < 0.01). Furthermore, the abundance of *Clostridium_sensu_stricto_1* (*p* < 0.01) and *Terrisporobacter* (*p* < 0.05) was significantly negatively correlated with average diarrhea rate.

## Discussion

### Effects of ZnL on growth performance of piglets

The core aim of this study was to assess the potential of dietary ZnL in the weaned piglets in comparison to a high-dose of conventional ZnO (1,500 ppm Zn). We comprehensively evaluated the growth performance, intestinal morphology, barrier function and gut microbiota of piglets. The results demonstrated that both 800 ppm dietary ZnL and 1,500 ppm ZnO significantly decreased diarrhea rate, which was in accordance with previous studies by Diao et al. (2021) and Yi et al. (2023). However, neither dietary ZnL at 800 ppm or 1,500 ppm ZnO had a significant impact on the growth performance, which might be attributed to the management level and the physiological condition of piglets. Tang et al. (2024) also reported that under normal physiological conditions, a ZnL-supplemented diet had little effect on growth performance, but it significantly enhanced ADG and reduced diarrhea frequency under oxidative stress. Based on the numerical improvements in growth performance and diarrhea indexes, the optimal level of ZnL was determined to be 800 ppm. This is in agreement with another study on organic zinc, which found that zinc aspartic acid chelate (750 ppm) could perform comparably to therapeutic ZnO (3,000 ppm) (Biswas et al., 2024). The consistency may be attributed to dietary Zn content quadratically affected growth performance of newly weaned piglets in the first 2 weeks (Hansen et al., 2022). The significant and large effect size differences in ADFI observed between CON+ and TRE1 may mainly be limited by the sample size and efficacy. Meanwhile, the small experimental scale (*n* = 4 replicates/group) could restrict the generalizability of ZnL’s efficacy in growth performance and diarrhea rate. Expanding the population size in subsequent trials is strongly recommended to confirm these preliminary observations.

Furthermore, on day 7 of the experiment, 1,500 ppm dietary ZnO significantly decreased the sensory scores of piglets’ skin and fur. This might be related to the fact that high-dose ZnO triggered Zn homeostatic mechanisms in weaned pigs and impaired Fe metabolism (Dalto et al., 2023a) and Cu metabolism (Dalto et al., 2023b). Additionally, frequent application of ZnO can lead to the accumulation of microelements in the pig production environment and the development of antibiotic resistance in pathogenic swine microflora (Pejsak et al., 2023). The European Union banned the use of ZnO in pig feed in 2022. These results suggest that ZnL can be a suitable alternative to ZnO, contributing to a lower diarrhea rate and improving the sensory indicators of weaned piglets. Adding ZnL (800 ppm) to the diet of weaned piglets while eliminating acidifiers could perform comparablely to high-dose ZnO (1,500 ppm). The costs of these two diets are similar, which means it is also feasible in large-scale pig farm production.

### Effects of ZnL on antioxidant capacity and intestinal health of piglets

Weaning disrupted the oxidative balance in piglets and caused oxidative injury, accompanied by a decrease in the plasma activity of SOD and GPx, and an increase in plasma MDA level after weaning (Yin et al., 2014). ZnL is an effective organic zinc source with antioxidant properties in mammals. Tang et al. (2020) demonstrated that ZnL could improve the antioxidant capacity and mitochondrial function in IPEC-J2 cells by activating the AMPK-Nrf2-p62 pathway under normal or oxidative stress conditions. Xiao et al. (2023) reported that supplementing with sodium alginate-coated nano zinc oxide promoted the levels of SOD and Cu/Zn-SOD in piglet serum, while ZnO addition decreased the MDA concentration. Huang et al. (2023) also found that low-dose zinc-loaded montmorillonite and high-dose conventional ZnO both significantly increased the activities of T-AOC and SOD, and decreased the MDA level in the serum and colonic mucosa of piglets. Similarly to previous findings, our study showed that both 800 ppm dietary ZnL and 1,500 ppm ZnO significantly increased the activities of SOD and Cu/Zn-SOD, which may help alleviate weaning stress.

The intestinal barrier function is a crucial index for evaluating piglet gut health, including intestinal histology and compact protein expression, etc. Organic zinc sources are more beneficial for piglet gut health. Pearce et al. (2015) found that dietary with amino acid Zn complex tended to mitigate heat stress-induced reduction in ileal villus height and improve intestinal integrity in growing pigs. Similarly, ZnL supplementation led to higher jejunal villus height and a higher villus height: crypt depth ratio in weaned piglets (Diao et al., 2021). Tang et al. (2024) also found that dietary ZnL supplementation increased jejunal and ileal villus heights in stressed piglets. In the present study, both 800 ppm ZnL and 1,500 ppm ZnO increased duodenal villus height, and ZnL increased villus height of jejunum and ileum, while ZnO increased villus width of jejunum and ileum. These results indicate that ZnL may serve as an alternative of ZnO to improve the intestinal structural integrity of piglets and reduce the dietary zinc level.

During the post-weaning period, impaired intestinal barrier function can cause diarrhea, growth retardation, or even death in piglets. Claudin-1, Occludin, and ZO-1 proteins are the most critical parts of the tight junction structure and play primary roles in maintaining intestinal barrier function (Tsukita et al., 2001). Zn strengthens the porcine jejunal epithelial barrier by reversibly tightening the paracellular route for inorganic ions and small solutes (Zakrzewski et al., 2017). Yu et al. (2022) reported that palygorskite clay-adsorbed nano-ZnO could effectively improve the intestinal barrier function of weanling piglets and potentially could replace high-dose ZnO at an appropriate dose of 700 mg/kg. Tang et al. (2024) found that ZnL improved intestinal morphology and ultrastructure by significantly increasing the expression level of the jejunal tight junction protein, zonula occludens-1. In our study, 800 ppm dietary ZnL significantly increased the mRNA expression of *CLDN-1* in the duodenum and ileum of piglets, while 1,500 ppm ZnO or 600 ppm ZnL had no significant effect. This suggest that the biological efficiency of zinc at 800 ppm ZnL may be higher than that at 1500 ppm ZnO. Although adminstration with 800 ppm ZnL numerically increased the mRNA expression of *OCLN* and *ZO-1* in the duodenum and ileum of piglets, these changes did not reach statistical significance. This might be attributed to limited experimental replicates or insufficient statistical power, and further experiments are needed.

### Effects of ZnL on cecal microbiota of piglets

Weaned piglets are in a transitional stage of internal organ development and external environment change, which often leads to intestinal disorders, damaged growth performance, and severe diarrhea (Sun Y. et al., 2022). Intestine chyme serves as the first line of defense, and the gut harbors a large number of microorganisms, which engage in multiple interactions affecting host health (Milani et al., 2017). The gut microbiota is susceptible to dietary modulation (Huting et al., 2021). ZnO has been widely used to relieve the post-weaning diarrhea. Xiao et al. (2023) found that dietary with ZnO reduced microbial *α*-diversity indexes such as Chao1, Shannon and Sobs in piglet feces. Luise et al. (2023) also found that ZnO treatment had lower α-diversity and a different microbial *β*-diversity of piglets. Similarly, the present study also found that 1,500 ppm dietary ZnO decreased microbial α-diversity indexes (Ace, Chao, Sobs and Shannon) and had a significantly different microbial β-diversity in piglets. Long et al. (2023) reported that porous ZnO particles in the piglet diet increased the microbial β-diversity index in both the ileum and colon, decreased microbial α-diversity in the ileum, and increased it in the colon. In this study, 800 ppm dietary ZnL had similar effects to 1,500 ppm ZnO, reducing the microbial α-diversity indexes Ace, Chao, Sobs and Shannon. Meanwhile, the microbial α-diversity indexes Ace, Chao, Sobs in the group with 600 ppm ZnL was higher than that with 800 ppm ZnL, indicating that the effect of ZnL on microbial α-diversity indexes might have a dose-effect.

Regarding the microbial composition, Yi et al. (2023) reported that piglets receiving ZnO diet had a higher abundance of the phylum *Bacteroidetes*, and genera *Prevotella*, and lower phylum *Firmicutes* and genera *Lactobacillus* in colonic contents. Peng et al. (2024) reported that 1,500 ppm dietary ZnO increased the abundance of *Firmicutes* in the jejunal and cecum contents, and decreased the abundance of *Proteobacteria* in the ileal and cecum contents of piglets. In the present study, 1,500 ppm dietary with ZnO increased the proportion of the phylum *Firmicutes*, and genera *Clostridium_sensu_stricto_1* and *Streptococcu*s, while decreasing the proportion of the phylum *Bacteroidota* and *Spirochaetota*, and genera *[Eubacterium]_ruminantium_group*. Organic zinc has higher bioavailability, Zhang et al. (2022) found that protein-chelated zinc (Zn-Pro) increased the relative abundance of *Lactobacillaceae* and *Lachnospiraceae* and decreased *Prevotellaceae* in piglets. Tang et al. (2024) reported that ZnL treatment altered the gut microbiota structure, significantly increasing the abundance of beneficial gut microbes, including *UCG_002*, *Ruminococcus*, *Rikenellaceae_RC9_gut_group*, *Christensenellaceae_R_7_group*, *Treponema*, *unclassified_Christensenellaceae*, and *unclassified_Erysipelotrichaceae*. The *Christensenellaceae_R_7_group* plays a critical role in modulating amino acid and lipid metabolism, thereby enhancing host metabolic homeostasis and exhibiting protective effects against obesity and related metabolic disorders (Waters and Ley, 2019). *Ruminococcus* are able to produce microbial carbohydrases and degrade complex plant material (Biddle et al., 2013). However, in our study, dietary with ZnL significantly decreased the abundance of genera *Christensenellaceae_R-7_group* and *Ruminococcus,* which might have a negative effect on the digestive capacity of piglets as the treatment diet has removed the addition of comprehensive acidifiers. There are limitations in this study to exploring the effects of ZnL on nutrient metabolism of weaned piglets, so further investigation is still needed.

### Associations between cecal microbiota and performance of piglets

We detected the top 10 bacteria at the genus level such as *Clostridium_sensu_stricto_1*, *Prevotellaceae_NK3B31_group*, *Prevotella _9*, *[Ruminococcus]_gauvreauii_group* and *Terrisporobacter*, by performing species- performance association analysis among groups. In the present study, the abundance of genera *Clostridium_sensu_stricto_1,* a potentially pathogenic bacteria associated with intestinal disorders (Fukuda et al., 2011), increased following dietary ZnO supplementation. The *clostridium_sensu_stricto_1* could reduce the diarrhea ratio by producing volatile fatty acids (VFAs), promoting pathogen adherence to the intestinal mucus barrier, and decreasing inflammatory factor contents (Forssten et al., 2011). Sun T. et al. (2022) found a significant negative correlation between the abundance of *Clostridium_sensu_stricto_1* bacteria and the diarrhea score of weaned piglets, and the FCR for 28-day production performance of weaned piglets. Similarly to previous studies, it was also found that the abundance of *Clostridium_sensu_stricto_1* was significantly negatively correlated with average diarrhea rate among groups. Moreover, the lipid metabolic profile in rat serum was highly correlated with *Blautia*, *Lachnoclostridium*, *Clostridium_sensu_stricto_1* and *Turicibacter* (Ni et al., 2023). Yan et al. (2024) reported that the abundance of *Clostridium* sensu stricto *1* in the gut microbiota was positively correlated with fatty acid degradation. In this study, it was showed that the abundance of *Clostridium* sensu stricto *1* of gut microbiota was positively correlated with MDA, which were increased by free fatty acid and high fat-diet (Mun et al., 2019).

*Turicibacter* was related to inflammation and immune cell balance (Song et al., 2022). Its abundance increased in both ZnO and ZnL groups, similar to previous studies (Xiao et al., 2023). In this study, the abundance of *Terrisporobacter* was significantly negatively correlated with the average diarrhea rate, which was in line with *Clostridium_sensu_stricto_1*. Additionally, Cao et al. (2022) reported that there was a positive correlation between intestinal GPx activity and the relative abundance of *Peptostreptococcaceae*. In this study, it was found that the abundance of *[Ruminococcus]_gauvreauii_group* was significantly positively correlated with serum GPx activity, and *Clostridium_sensu_stricto_1* was significantly positively correlated with serum SOD activity. These correlation analyses suggested that the differential microbes upregulated by ZnO and ZnL administration were highly positively associated with antioxidant and significantly negatively correlated with diarrhea rate, indicating an improvement of intestinal health and growth performance via the crosstalk between gut microbes and dietary with ZnO or ZnL.

## Conclusion

In summary, the present research indicated that dietary with 800 ppm Zn/kg from ZnL could reduce diarrhea rate, improve intestinal morphology and enhance antioxidant capacity of weaned piglets, even show more pronounced effects on intestinal barrier function compared with traditional ZnO. Furthermore, both ZnO and ZnL adjusted the diversity and structure of gut microbiota, which was significantly correlated with diarrhea rate and serum antioxidant capacity. Altogether, 800 ppm ZnL demonstrated comparable efficacy to 1,500 ppm high-dose ZnO in enhancing anti-diarrheal effects and antioxidant capacity in weaned piglets, potentially mediated through modulation of gut microbial *α*-diversity and composition. So mention as ZnL shows promise as a ZnO alternative, though further large-scale trials and cost analyses are needed to validate its practical application.

## Data Availability

The original contributions presented in the study are publicly available. This data can be found here: https://www.ncbi.nlm.nih.gov, accession number PRJNA1258601.

## Footnotes

^1^ www.majorbio.com

^2^ http://www.mothur.org/wiki/Calculators
